# Ischemic postconditioning influences electron transport chain protein turnover in Langendorff-perfused rat hearts

**DOI:** 10.7717/peerj.1706

**Published:** 2016-02-16

**Authors:** Song Cao, Yun Liu, Haiying Wang, Xiaowen Mao, Jincong Chen, Jiming Liu, Zhengyuan Xia, Lin Zhang, Xingkui Liu, Tian Yu

**Affiliations:** 1 Department of Anesthesiology, Zunyi Medical College, Zunyi, China; 2 Guizhou Key Laboratory of Anesthesia and Organ Protection, Zunyi Medical College, Zunyi, China; 3 Department of Pain Medicine, Affiliated Hospital of Zunyi Medical College, Zunyi, China; 4 Research Center for Medicine & Biology, Zunyi Medical College, Zunyi, China; 5 Department of Anesthesiology, The University of Hong Kong, Hong Kong, China

**Keywords:** Ischemia-reperfusion (I/R) injury, Mitochondrial ATP-sensitive potassium channel (mitoKATP), Energy homeostasis, Postconditioning, Comparative proteomics, Electron transport chain

## Abstract

Ischemia postconditioning (IPo) is a promising strategy in reducing myocardial ischemia reperfusion (I/R) injury (MIRI), but its specific molecular mechanism is incompletely understood. Langendorff-perfused isolated rat hearts were subjected to global I/R and received IPo in the absence or presence of the mitochondrial ATP-sensitive potassium channel (mitoKATP) blocker 5-hydroxydecanoate (5-HD). Myocardial mitochondria were extracted and mitochondrial comparative proteomics was analyzed. IPo significantly reduces post-ischemic myocardial infarction and improved cardiac function in I/R rat hearts, while 5-HD basically cancelled IPo’s myocardial protective effect. Joint application of two-dimensional polyacrylamide gel electrophoresis (2DE) and MALDI-TOF MS identified eight differentially expressed proteins between groups. Expression of cardiac succinate dehydrogenase (ubiquinone) flavoprotein subunit (SDHA) increased more than two-fold after I/R, while IPo led to overexpression of dihydrolipoyl dehydrogenase (DLD), NADH dehydrogenase (ubiquinone) flavoprotein 1 and isoform CRA_b (NDUFV1). When the mitoKATP was blocked, MICOS complex subunit Mic60 (IMMT) and Stress-70 protein (Grp75) were over expressed, while DLDH, ATPase subunit A (ATPA) and rCG44606 were decreased. Seven of the differential proteins belong to electron transport chain (ETC) or metabolism regulating proteins, and five of them were induced by closing mitoKATP in I/R hearts. We thus conclude that IPo’s myocardial protective effect relies on energy homeostasis regulation. DLD, SDHA, NDUFV1, Grp75, ATPA and rCG44606 may contribute to IPo’s cardial protective effect.

## Introduction

Although percutaneous coronary intervention (PCI) and coronary artery bypass graft (CABG) are effective in mitigating myocardial infarction (MI), myocardial ischemia reperfusion injury (MIRI) induced by reperfusion is always inevitable (Yellon & Hausenloy, 2007). Besides the exiting discovery of the protective effect of ischemia preconditioning (IPC) by Murry, Jennings & Reimer (1986) and Zhao and colleagues (2003) reported that repetitive brief episodes of reperfusion/ischemia applied at the onset of prolonged reperfusion (i.e., ischemia postconditioning , IPo), can also protect the heart against MIRI. Since then, accumulating literatures reported that IPo has protective effects against MIRI (Darling et al., 2005; Khan et al., 2014; Kin et al., 2004; Tang et al., 2006). The beneficial action of IPo is considered to be secondary to its effect on various cellular mechanisms: Janus signal transducer and activator system, reduction of the reactive oxygen or nitrogen species generation, and attenuation of intracellular calcium overload (Heusch, 2013; Heusch, 2015b). All of these mediators decrease lethal reperfusion injury and reduce apoptosis of cardiomyocytes (Heusch, 2013), but IPo’s effects remains controversial in human. Some studies reported that it exerted cardioprotection (Staat et al., 2005; Thibault et al., 2008), while others observed negative results (Bodi et al., 2014; Hahn et al., 2013; Lønborg et al., 2010; Tarantini et al., 2012). In spite of its reproducibility and cost-effectiveness in decreasing reperfusion injury, there are questions to be addressed before IPo can be used routinely in clinical settings (Hansen, Thibault & Abdulla, 2010; Heusch, 2015a; Khan et al., 2014). Meanwhile, the mechanisms by which IPo attenuates cardiac I/R injury need to be further elucidated before its clinical use (Heusch, 2015a).

Mitochondria not only energize cellular activities but also generate reactive oxygen species (ROS) in cardiomyocytes via one-electron transfer reactions. In fact, mitochondrion is critical to reperfusion injury because of the damage sustained during ischemia. Approaches targeting mitochondrion, e.g., the succinate dehydrogenase (SDH, mitochondrial complex II) of electron transport chain (ETC) (Chouchani et al., 2014) , the ND3 subunit of complex I (Chouchani et al., 2013) or the STAT3 signaling in mitochondria (Heusch, 2015a), can reduce MIRI. Mitochondrial ATP-sensitive potassium channel (mitoKATP), which anchors on the inner membrane of mitochondria, is a common end effector of many protective stimuli in MIRI (Testai et al., 2015). Pharmacological inhibition of the mitoKATP, e.g., with its selective antagonist 5-hydroxydecanoate (5-HD) in early reperfusion abolished the infarct-limiting effect of IPo (Gross, 1995; Mykytenko et al., 2008; Yang et al., 2004), while opening it reduced excessive generation of ROS and the resultant Ca^2+^ overload (Jin et al., 2012). We found that opening of mitoKATP reduced MIRI (Yang & Yu, 2010; Yu et al., 2011; Yu et al., 2001) and improved energy homeostasis in anoxia/reoxygenation (A/R) cardiomyocytes (Cao et al., 2015).

We hypothesize that IPo’s myocardial protective effect is resulted from regulation of myocardial mitochondrial proteins. In addition, we evaluated mitoKATP’s contribution in IPo process. Mitochondrial proteomics was performed in a MIRI rat model to establish cardioprotective mechanisms of IPo and the role of mitoKATP. We identified eight differential proteins, most of which are components of electron transport chain (ETC) complexes.

## Material and Methods

### Animals

Sprague-Dawley rats (male, 250–300 g) were from the laboratory animal center of the Third Military Medical University (Chongqing, China), and maintained in specific pathogen-free (SPF) animal facilities in Zunyi Medical College. They were given 12 h light/dark cycles and free access to rat chow and water. All experimental procedures were performed according to the “Guide for the Care and Use of Laboratory Animals” in China and approved by the Experimental Animal Care and Use Committee of Zunyi Medical College.

### Materials

Acetonitrile, trifluoroacetic acid (TFA), K_3_Fe(CN)_6_, HCCA, 5-hydroxydecanote (5-HD), Nycodenz and urea were purchased from Sigma (St. Louis, MO, USA). Bromophenol blue, acrylamide, CHAPS, immobilized pH gradient (IPG) strips, two-dimensional polyacrylamide gel electrophoresis (2DE) Quant kit, 2DE Clean-up Kit, polyvinylidene fluoride membrane and low melting point agarose were purchased from Bio-Rad (Hercules, CA, USA). Glycerol, glycine, ammonium persulfate, N, N′-methylene acrylamide, SDS, Tris, EDTA, sucrose and mannitol were supplied by Amresco (Solon, OH, USA). Secondary antibodies and anti-GRP75, anti-NDUFV1, anti-DLD and anti-cytochrome oxidase IV (COX IV) primary antibodies were obtained from Abcam (Cambridge, UK). TEMED was from Amersham (UK). Butanol was from Merck (Darmstadt, Germany). Trypsin was from Promega (Madison, WI, USA). Pentobarbital sodium and Tween 20 were both from Solarbio (Beijing, China). All other unlisted reagents were of analytical grade or guaranteed reagents.

### Equipments and software

The main equipments included 2DE electrophoresis system (Bio-Rad, Hercules, CA, USA), gel scanner (Epson, Tokyo, Japan), MALDI-TOF-TOF mass spectrometer (Ultraflex III, Bruker, Germany), ultra-high speed centrifuge (Beckman, Brea, CA, USA), PowerLab system (AD instrument, Australia), transmission electron microscope (TEM, HITACHI, Tokyo, Japan), tissue homogenizer (IKA, Germany), Odyssey infrared imaging system (LI-COR, Lincoln, NE, USA). Software contained PDQuest advanced 8.0 2DE analysis software (Bio-Rad, Hercules, CA, USA), data explorer, mass spectrometry analysis software, Mascot Distiller mass spectrometry signal peak recognition software, Mascot peptide mass fingerprinting database query software (Applied Biosystem; Life Technologies, Carlsbad, CA, USA); Mascot TOF/MS database equery software (Matrix Science, London, UK) and Odyssey infrared imaging analysis software (LI-COR, Lincoln, NE, USA).

### Reperfusion protocol

**Figure 1:**
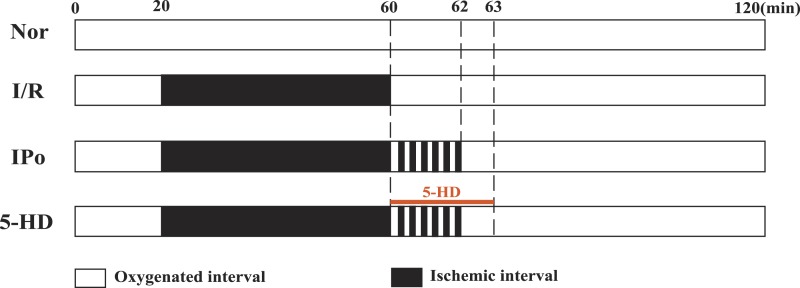
Reperfusion protocol. Rats were allocated into 4 groups: normal (Nor), I/R (I/R), IPo (IPo) and 5-hydroxydecanoate + IPo (5-HD). Rat hearts were equilibrated for at least 20 min before the application of above protocols. Excluding the Nor group, all hearts underwent ischemia for 40 min (indicated with black area). The I/R group were exposed to 40 min ischemia alone. The hearts in IPo group underwent IPo (palisade white and black bars) alone while 5-HD was administrated in the first 3 min of reperfusion in 5-HD group.

Reperfusion protocol was prepared as we previously did (Yu et al., 2011; Yu et al., 2001). Twenty-four rats were randomly divided into four groups (normal (Nor), I/R (I/R), IPo (IPo) and 5-hydroxydecanoate + IPo (5-HD); *n* = 6). Rats were intraperitoneally injected 30 mg/kg pentobarbital sodium and 250 U/kg heparin. Then, the hearts were quickly removed and connected to a Langendorff perfusion system. Hearts were perfused with 37 °C Krebs–Henseleit (K–H) buffer containing (in mM): 118.0 NaCl, 4.75 KCl, 2.50 CaCl_2_, 24.80 NaHCO_3_, 1.19 MgCl_2_∙6H_2_O, 1.19 KH_2_PO_4_, 11.1 Glucose; pH 7.40; bubbled for 10 min before perfusion with 5% CO_2_ and 95% O_2_) at 5.8 kPa (perfusion pressure) for 20 min before equilibration. Perfusion protocols were shown in Fig. 1. Group Nor was perfused with K-H solution for 120 min without induced MIRI. The other 3 groups were perfused with K-H solution for 20 min for equilibration. Next, St. Thomas solution (NaCl 110, KCl 16, CaCl_2_ 1.2, MgCl_2_ 16, NaHCO_3_ 10 (in mM); pH 7.80) was perfused to stop the heart beating to induce a 40 min global ischemia. After 40 min cardiac arrest, group I/R was perfused with the K–H solution for 60 min. For group IPo, hearts underwent IPo (6 cycles of reperfusion for 10 s, and arrest for 10 sec) first, followed by K–H solution perfusion for 58 min. Group 5-HD received IPo, at the beginning of IPo, 100 µM 5-hydroxydecanoate (5-HD, mitoKATP blocker) was given via K-H perfusion solution in the first 3 min, then followed by another 57 min perfusion. The cardiac function parameters (i.e., heart rate (HR), the maximum rate of rise of intraventricular pressure (*dp*∕*dt*_max_), left ventricular developed pressure (LVDP) and left ventricular end-diastolic pressure (LVDEP)) of each group were recorded with PowerLab system (AD Instrument, Australia). Parameters at the end of equilibration (T1) and end of reperfusion (T2) were statistically analyzed. If premature systoles < 2/min, HR > 250 bpm and LVDP > 80 mmHg by the end of T1, ventricular tissue of each heart was collected at the end of reperfusion.

### Myocardial infarct size measurement

At the end of reperfusion, 1% 2,3,5-triphenyl-tetrazolium chloride (TTC, in phosphate buffer at pH 7.4) was pumped into hearts. When the epicardial surface turned deep red, hearts were fixed in 10% formalin. The infarcted areas (white) and viable areas (red) for each slice were traced and digitized using a computerized planimetry technique (SigmaScan; Systat Software, San Jose, CA, USA). The ratio of infarct size /total area was employed to evaluate the infarct size differences among groups.

### Electron microscopy of the Langendorff myocardium

Electron microscopy was conducted as we previously reported (Yang & Yu, 2010). Briefly, at the end of reperfusion, one cubic millimeter of myocardium of the left ventricle was fixed in 0.25% glutaraldehyde and 3% paraformaldehyde. Then, samples were mounted with 1% osmic acid, dehydrated with acetone, and embedded with ethoxyline 618. Ultrathin myocardium sections were made and photographed with a transmission electron microscope (HITACHI-H7500, Japan). Ultrastructural damage was assayed with Flameng’s score method (Flameng et al., 1980). For each sample, 20 mitochondria from five fields were randomly selected. The criteria for the judgement of mitochondrial damage from grade 0 to 4 were as follows: grade 0, mitochondrial granules were integrated, with normal structure; grade 1, mitochondrial granules were lost, but with normal structure; grade 2, mitochondrial granules were lost, and with swelling mitochondria and limpid matrix; grade 3, mitochondrial granules were lost and with limpid matrix and ruptured cristae; grade 4, mitochondrial granules were lost, with ruptured cristae but short of complete inner and external membranes.

### Myocardial mitochondria extraction

The preparation of high purity intact mitochondria is the first crucial step for the mitochondrial comparative proteomics, which effectively excludes cytoplasmic and cytomembrane proteins. The mitochondria were prepared as Kim et al. (2006) and Jiang et al. (2005) reported but with some necessary modifications. Firstly, cardiac tissues were homogenized in mitochondrial extraction buffer (MEB) that contained 0.21 M mannitol, 0.07 M sucrose, 10 mM Tris base and 1 mM EDTA at pH 7.35–7.45. The resultant homogenate was centrifuged at 1,500 g for 10 min at 4 °C. Afterwards, the supernatant was centrifuged at 10,000 g for 10 min. Then the mitochondria-enriched pellet was homogenized in 1.2 mL of 25% Nycodenz in MEB for the second purification on a Nycodenz density gradient. This gradient was carefully prepared by stepwise layering of 0.5 mL of 34%, 0.8 mL of 30%, the 1.2 mL mitochondria-enriched 25%, 0.8 mL of 23% and 0.3 mL of 20% Nycodenz solutions in a tube and centrifuged at 100,000 g for 60 min at 4 °C. The white band in the tube at the interface of the 23% and 25% Nycodenz solutions was collected, diluted with MEB and centrifuged twice at 12,000 g for 10 min. The purity of the mitochondria was evaluated by transmission electron microscope (HITACHI-H7500; Hitachi, Tokyo, Japan).

### The 2DE of mitochondrial proteins

The purified mitochondria were dissolved in RIPA buffer and kept at room temperature for 10 min. Mitochondrial protein was extracted after ultrasonication and centrifugation. The protein was purified with Clean-up Kit (Bio-Rad, Hercules, CA, USA) and quantified with RC DC™ (Reducing agent Compatible & Detergent Compatible) method using a 2DE Quant kit (Bio-Rad, Hercules, CA, USA). Two-dimensional polyacrylamide gel electrophoresis (2DE) was performed as previously described (Kim et al., 2006) with some modifications. The 24 cm, pH = 5–8 immobilized pH gradient (IPG) strips were rehydrated for 14 h at 50 V in 500 mL of an isoelectric focusing (IEF) solution that contained approximately 500 mg mitochondrial protein. IEF was carried out as follows: 250 V for 1 h, 1,000 V for 3 h, 4,000 V for 3 h, 10,000 V incremented to 80,000 V/h finally. The IPG strips were placed in 8 mL of an equilibration solution (375 mM Tris–HCl, pH 8.8, containing 20% glycerol, 2% SDS, 6 M urea and 0.001% bromophenol blue) containing 2% (w/v) DTT for the first equilibration step (15 min) and 2.5% (w/v) iodoacetamide for the second equilibration step (15 min). The 2DE separation was performed in the PROTEIN plus Dodeca cell (Bio-Rad, Hercules, CA, USA). IPG strips were loaded onto 12% SDS-PAGE gels, running buffer (25 mM Tris, 192 mM glycine, 0.1% (w/v) SDS) was added, a 200 V constant voltage was applied for 6 h. Afterwards, gels were stained with silver nitrate and scanned with a flat bed scanner (Epson, Tokyo, Japan). Images were analyzed using commercially available software (PDQuest advanced 8.0; Bio-Rad, Hercules, CA, USA). A more than ±200% expression change was set as the threshold to detect differential proteins between two groups.

### Protein identification

MS fingerprinting was performed as Rosenfeld et al., (1992) described. The silver nitrate stained differential proteins were excised and digested with trypsin. Gel granules containing target proteins were destained with sodium thiosulfate and potassium ferricyanide. After washing with ACN, gels were dried in a vacuum centrifuge. Dried gels were rehydrated in 100 mM NH_4_HCO_3_ that contained 12.5 mg/L sequencing-grade trypsin (Promega, Madison, WI, USA). Then the mixture were incubated overnight at 37 °C. Peptides were extracted twice with 50 mM NH_4_HCO_3_:ACN (1:1, v/v) mixture. Three µL of the peptide solution was applied to a target disk to evaporate before 0.1 µL matrix solution (4 mg/mL in 70% ACN/30% 0.1% TFA, v/v) and 0.1 µL calibration standard were added. Spectra were obtained using a Ultraflex III MALDI TOF/TOF mass spectrometer (Bruker, Bremen, Germany). BioTools 3.0 (Bruker, Bremen, Germany) was employed and MASCOT (Matrix Science, London, UK) was used as the database search algorithm to identify proteins via peptide mass fingerprinting (PMF). NCBInr was chosen as the sequence database. The MASCOT search parameters were: database: NCBInr 20111105 (15916306 sequences; 5467648827 residues); taxonomy: rattus (67400 sequences); type of search: MS/MS ion search; enzyme: trypsin; fixed modifications: carbamidomethyl (C); variable modifications: oxidation (*M*); mass values: monoisotopic; protein mass: unrestricted; peptide mass tolerance: ±75 ppm; fragment mass tolerance: ±0.5 Da; max missed cleavages: 1; instrument type: MALDI-TOF-TOF. The Probability Based Mowse Score is −10*Log(*P*), where *P* is the probability of the observed match. Scores greater than 61 are significant (*P* < 0.05).

### Western Blot validation

Twenty microgram of mitochondrial protein was separated by 12% SDS-PAGE. The target proteins were transferred to polyvinylidene fluoride (PVDF) membranes, which were blocked overnight in TBS (20 mM Tris and 150 mM NaCl, pH 8.0) containing nonfat milk powder. Then membranes were probed with 1 mg/mL antibodies of DLD, GRP75, NDUFV1 and COX IV for 1 h. PVDF membranes were incubated with horseradish peroxidase-conjugated secondary antibody (1:2,000) for 1 h. Immunoreactivity was visualized by Odyssey infrared imaging system (LI-COR, Lincoln, NE, USA).

### Statistical analysis

Data were displayed as mean ± standard deviation (SD). SPSS 17.0 (IBM, USA) was used for statistical analyses. Differences between groups were analyzed by one-way analysis of variance (ANOVA). LSD or Dunnett’s T3 method was used to make comparisons between two groups. *P* values less than 0.05 were considered statistically significant.

## Results

### Hemodynamic parameters and infarct size

**Figure 2:**
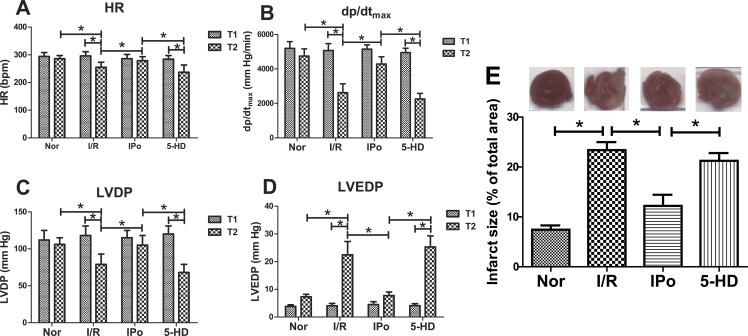
Comparison of hemodynamic parameters and infarct size. IPo effectively reduced myocardial dysfunction (A–D) and infarct size (E) induced by MIRI while 5-HD partially conteracted IPo’s effects on hemodynamic parameters and infarct size. *n* = 6 (A–D) or 4 (E) in each group. ^∗^*P* < 0.05.

IPo effectively reduced MIRI induced myocardial hemodynamic dysfunction (Figs. 2A–2D) and myocardium infarction (Fig. 2E), which was partially cancelled by 5-HD treatment. There was no significant difference (*P* > 0.05) for HR (Fig. 2A), *dp*∕*dt*_max_ (Fig. 2B), LVDP (Fig. 2C) and LVDEP (Fig. 2D) among 4 groups at the end of equilibration (T1), but heart function (shown as HR, *dp*∕*dt*_max_ and LVDP) of the Nor group and the IPo group at the end of perfusion (T2) were significantly better than those in the I/R group and the 5-HD group (*P* < 0.05).

### Electron microscopy

**Figure 3:**
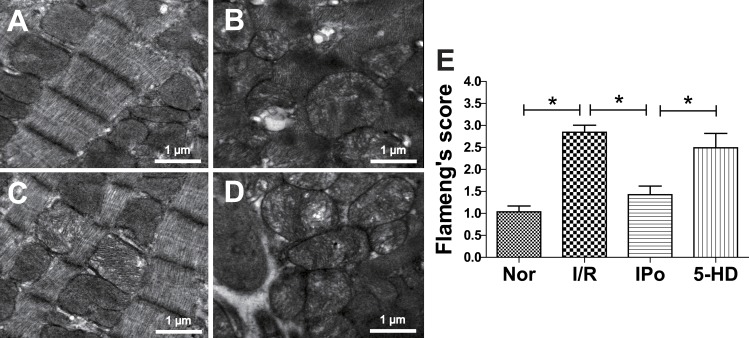
Transmission electron microscopy analysis of myocardium damage. TEM of Langendorff-perfused myocardium at the end of reperfusion in Control group (A), I/R group (B), IPo group (C) and 5-HD group (D). (E) Flameng’s score of mitochondria from electron microscopy. *n* = 100 for each group, ^∗^*P* < 0.05.

In the I/R group, myocardial fibers were in disorder, mitochondria were moderately swollen and cristae were disposed with vacuolar degeneration and rupture (Fig. 3B). The IPo group showed much better morphology: myocardial fibers were neatly arranged, and mitochondria were only slightly swollen, with intact appearance (Fig. 3C). However, when the mitochondrial potassium channel blocker 5-HD was added, the damage was intensified (Fig. 3D). Quantization of mitochondrial lesion was determined with Flameng’s score method as is shown in Fig. 3E. Flameng’s score was more than doubled in the I/R group, from 1.04 to 2.84, and dropped to 1.43 when IPo was applied. 5-HD partially but significantly offset IPo’s effect.

**Figure 4:**
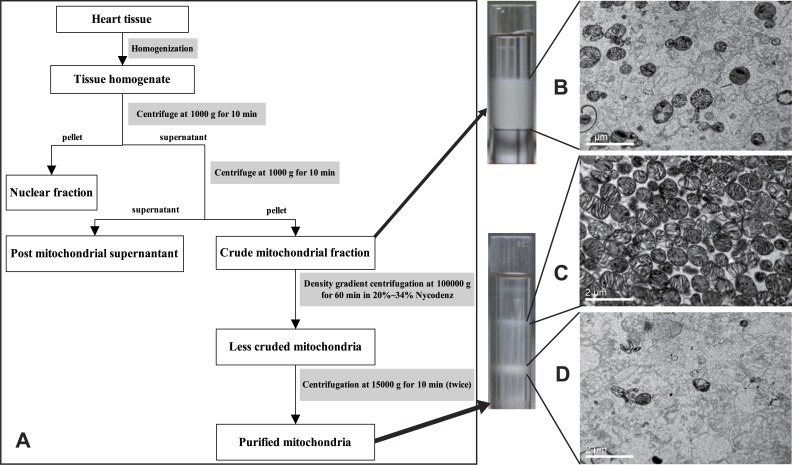
Flow chart of mitochondria preparation. Pure mitochondria are collected with Nycodenz density gradient centrifugation (A) and representative transmission electron micrograph of the isolated purified mitochondria (C). (B) Crude mitochondria. (C) Sample came from 23% to 25% layer of Nycodenz. (D) Sample isolated from the 25% to 30% layer Nycodenz. Scale bars represent 2 µm.

### Purity and integrity detection of myocardial mitochondria

The purity of mitochondria was evaluated by a transmission electron microscope (HITACHI-H7500; Hitachi, Tokyo, Japan), which showed that high purity mitochondria were obtained (Fig. 4C). Representative transmission electron micrographs of the isolated mitochondria are shown in Fig. 4. The mitochondria were extracted intact, with no swell or rupture and the cristae were integrated and organized. Besides intact mitochondria, there was little impurity element in fields of view.

### Changes in mitochondrial protein expression

**Figure 5:**
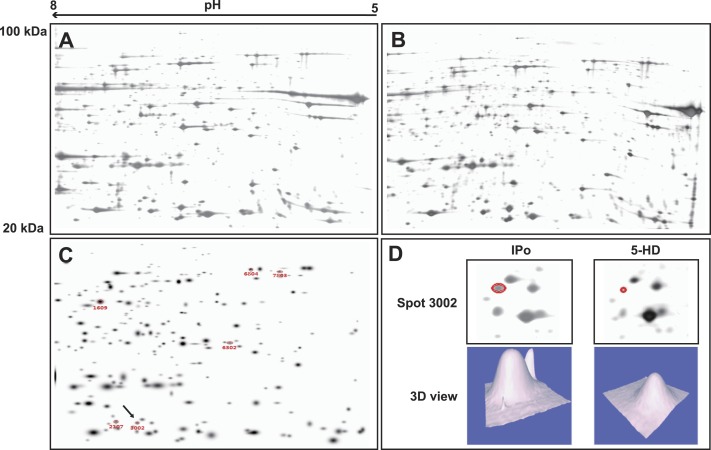
Proteomic comparison of differential proteins from rat mitochondria. Langendorff hearts were treated with 5-HD (A) or IPo (B) after I/R insult. Total mitochondrial protein samples were separated using linear IPG strips (24 cm, pH 5–8), and stained with silver nitrate. (C) Protein spots with differential parts per million (PPM) value were annotated with numbers in an image synthesized by PDQuest software. Protein in spot 3002 (detected as ATPA by MASCOT peptide mass fingerprint (PMF) and indicated with a black arrow) was detected as a differential protein by PDQuest. (D) Enlarged 2DE gel images and 3D view showing the 5-HD- and IPo-dependent protein expression changes of ATPA.

Mitochondrial proteins from Nor, I/R IPo and 5-HD were prepared as aforementioned. For the comparison of expression levels of mitochondrial proteins in the four groups, 2DE was performed. Differential expression in 2DE gels were analyzed by PDQuest advanced 8.0. Protein quantity of each spot was normalized to the total quantity of all valid spots and is expressed as parts per million (PPM). There was no significant difference for average protein spot number and matching ratio among the four groups (*P* > 0.05). Between Nor and I/R, I/R and IPo, IPo and 5-HD, 13 proteins in total were differentially expressed (with a change of more than ±200%). Differential proteins were excised from the gel and subsequent tryptic digests were analyzed by MALDI-TOF MS. Eight of them were successfully identified. The information of these differential proteins is shown in Table 1. The expression patterns of protein spot 3002 (number given by PDQuest advanced 8.0, identified by MALDI-TOF MS as H^+^-transporting ATP synthase subunit A (ATPA)) is presented (Fig. 5) as an example to illustrate the process of screening and identification of differential proteins.

**Table 1:** Information list of differential proteins.

Comparison	Protein name	Abbreviation	Calculated PI	Calculated Mr	SSP^a^	NCBI no.	AA% coverage^b^	Score^c^	Fold change^d^	*P* value^e^
Nor vs I/R	Succinate dehydrogenase [ubiquinone] flavoprotein subunit, mitochondrial	SDHA	6.75	72,596	5,805	gi|18426858	23%	326	2.40 ± 0.24	0.002
I/R vs IPo	NADH dehydrogenase (ubiquinone) flavoprotein 1, isoform CRA_b	NDUFV1	5.89	36,844	1,507	gi|149061921	32%	176	2.3 ± 0.53	0.005
	Dihydrolipoyl dehydrogenase, mitochondrial precursor	DLD	7.96	54,574	2,706	gi|40786469	14%	62	2.58 ± 0.49	0.008
IPo vs 5-HD	Dihydrolipoyl dehydrogenase, mitochondrial precursor	DLD	7.96	54,574	1,609	gi|40786469	19%	156	−2.96 ± 0.83	0.003
	rCG44606	rCG44606	9.13	40,522	2,107	gi|149059002	21%	134	−2.72 ± 0.98	0.003
	ATP synthase subunit alpha, mitochondrial	ATPA	7.03	25,639	3,002	gi|57029	44%	185	−2.02 ± 0.29	0.003
	MICOS complex subunit Mic60 (mitochondrial inner membrane protein)	IMMT	5.57	67,477	6,804	gi|77917546	22%	253	2.15 ± 0.82	0.012
	Stress-70 protein, mitochondrial (GRP-75)	GRP75	5.87	73,984	7,803	gi|1000439	15%	194	2.54 ± 0.84	0.001

**Notes.**

a Protein spot numbers given by PDQuest.

b Differential proteins’ amino acid coverage rate in reference proteins in NCBInr.

c By MALDI-TOF MS analysis.

d Expression change (more than ±200%), *n* = 4. For the ratio of Fold change of A vs B, “+” means B is overexpressed, “−” means B is down-regulated.

e Statistics of expression level expressed as parts per million (PPM) with unpaired *t* test, *n* = 4.

### Western Blot validation

**Figure 6:**
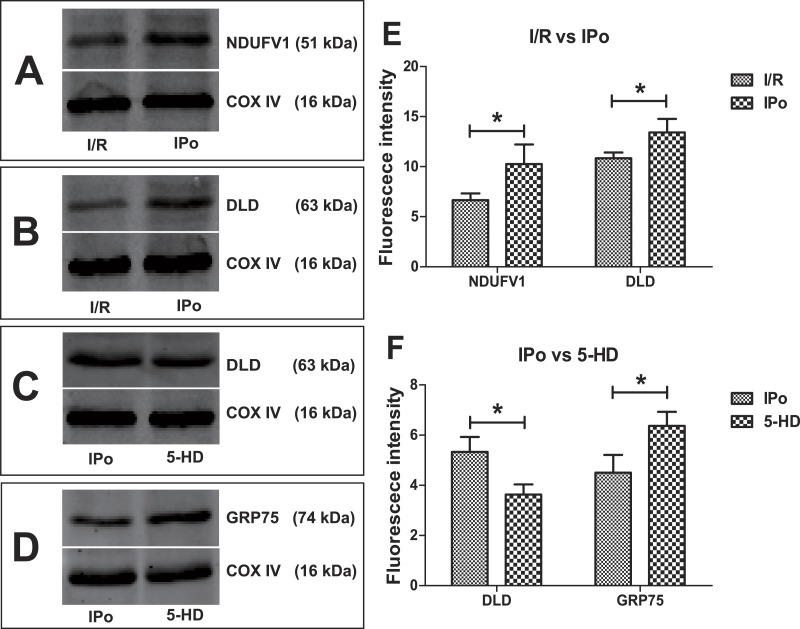
Expression trends of DLD, GRP5 and NDUFV1. Compared with the I/R group, NDUFV1 and DLD increased relatively in IPo group (A, B, E); Compared with the IPo group, GRP75 up-regulated while DLD down-regulated in the 5-HD group (C, D, F). Three replicates for each group. ^∗^*P* < 0.05.

To validate the degree of accuracy of 2DE, four interesting proteins were randomly selected and subjected to Western Blot. They were dihydrolipoyl dehydrogenase, mitochondrial precursor (DLD), stress-70 protein, mitochondrial (GRP75), NADH dehydrogenase (ubiquinone) flavoprotein 1, isoform CRA_b (NDUFV1). The Western Blot data (normalized to COX IV, Fig. 6) confirmed the reliability of 2DE and PMF results. Compared with the I/R group, the expression levels of DLD and NDUFV1 increased relatively in IPo group (Fig. 6E), while mitoKATP blockade with 5-HD resulted in significant up-regulation of GRP75 but down-regulation of DLD as compared to IPo group (*P* < 0.05, 5-HD vs. IPo, Fig. 6F).

## Discussion

Comparative proteomics focuses on differentially expressed proteins, which are the direct manipulator of various pathological and physiological states. Kim et al. (2006) employed proteomics to analyze differential proteins in mitochondrial fractions from normal, ischemia-reperfusion (I/R) and IPC treated rabbit hearts and identified some potential biomarkers. We investigated the effects of IPo on I/R rat hearts, especially IPo’s influences on cardiac mitochondrial proteomics. Eight mitochondrial proteins differentially expressed after I/R, IPo or mitoKATP blocking treatment (Table 1).

All of the eight proteins—succinate dehydrogenase [ubiquinone] flavoprotein subunit, mitochondrial (SDHA), DLD (differential protein from both I/R vs. IPo & 5-HD vs. IPo), NDUFV1, MICOS complex subunit Mic60 (mitochondrial inner membrane protein, IMMT), GRP75, ATPA and rCG44606, belong to the ETC or are energy metabolism related. Given that devastating effect of pathogenic mutations of mitochondria (with lower OXPHOS enzyme efficiency) has been shown to be a major mechanism leading to cardiomyopathies (Alston et al., 2015; Marín-García, 2001), and lack of energy homeostasis exacerbated post-ischemic myocardial injury and dysfunction (Cao et al., 2015), we postulate that alterations of mitochondrial protein turnover, may partially represent molecular mechanisms of IPo’s effects.

Dihydrolipoamide dehydrogenase (DLD) is a flavin dependent mitochondrial enzyme, which is essential for energy metabolism. In this study, DLD was up-regulated by IPo but IPo mediated this up-regulation of DLD was compromised by 5-HD. DLD is required by at least five multienzyme complexes (Babady et al., 2007). In the mitochondrial matrix, DLD is an E3 component of the *α*-ketoglutarate-pyruvate and amino acid-dehydrogenase complexes. It’s a part of the glycine cleavage system too. In these complexes, DLD is responsible for converting dihydrolipoic acid and NAD^+^ into lipoic acid and NADH (Carothers, Pons & Patel, 1989). In addition, DLD plays an antioxidant role. It scavenges nitric oxide and reduces ubiquinone to ubiquinol (Igamberdiev et al., 2004; Xia et al., 2001). Thus, DLD represents a highly versatile oxidoreductase with multiple critical roles in energy metabolism and redox balance (Bryk et al., 2002; Klyachko et al., 2005). Our data shows that IPo’s myocardial protective effect is consistent with the up-regulation of DLD. DLD could had enhanced energy product and accelerated scavenging of oxidants. This provides evidence that DLD could be a potential target in defeating MIRI.

Energy is often released in the form of H^+^, moving down an electrochemical gradient, such as from the lumen into the stroma of chloroplasts or from the inter-membrane space into the matrix in mitochondria. Three of the proteins, ATPA, NDUFV1 and SDHA are components of ETC complexes. ATPA belongs to complex IV, NDUFV1 attributes to complex I while SDHA is a part of complex II. ATPase provides energy for cardiomyocytes by synthesizing ATP through the reaction ADP + Pi → ATP, where ADP and Pi are joined together by ATPase. NADH dehydrogenase [ubiquinone] flavoprotein 1 (NDUFV1) is the 51 kD subunit of complex I (NADH:ubiquinone oxidoreductase), which is the front door of the mitochondrial respiratory chain (Ali et al., 1993). SDHA, which was up-regulated more than 2-fold at the end of I/R in the current study, participates in both tricarboxylic acid (TCA) cycle and ETC. It is responsible for the transfer of electrons from succinate to ubiquinone (succinate + ubiquinone → fumarate + ubiquinol). Recently, Chouchani et al. (2014) found that succinate accumulated for the reversal of SDH during ischemic period, and the accumulated succinate was re-oxidized by SDH in the reperfusion period, resulting in extensive ROS generation at complex I. Furthermore, decreasing succinate accumulation by pharmacological inhibition SDH was sufficient to ameliorate MIRI in rat. It’s also reported that inhibition SDH with malonate at the onset of reperfusion reduced infarct size in mice hearts through reduction ROS production (Valls-Lacalle et al., 2015). Up-regulation of SDH in I/R group (compared with Nor) may act as a compensation for the deficit of energy. In addition, this may also contributed to MIRI by producing excessive ROS during the reperfusion period. The normal SDH activity, both the reversal activity in ischemic phase and forward activity in reperfusion period, contributes to the production of ROS, and the MIRI too. Theoretically, a moderate down-regulation (e.g., miRNA interference or siRNA technique, test with pilot animal study) of SDH (or SDHA) may be a strategy to defeat MIRI. After IPo treatment, NDUFV1 upregulated more than two-fold, while mitoKATP blocker down-regulated ATPA by more than 200%, which indicated that NDUFV1 and ATPA could be served as surrogates of IPo in defeating MIRI.

To date, function of rCG44606 is not defined. We blasted its sequence in NCBI Blast against the Rattus norvegicus protein sequences in NCBInr and found it matches well (with a 92% identical amino acid rate) with that of creatine kinase (CK) *S*-type, mitochondrial (Rattus norvegicus) (NP_001121124.1). A detailed comparison between them is displayed in Table 2. Interestingly, their protein sequences are 100% identical for amino acid 1–224. The calculated pI and Mr of rCG44606 is 9.13 and 40.5 kDa respectively, which didn’t match well with its actual value in gels (Fig. 5). Considering PMF may give false positive results, rCG44606 could be the conventional creatine kinase *S*-type (NP_001121124.1, calculated pI 8.64, Mr 47473). To confirm this, additional mass spectrometry evidence is required. For example, a peptide that is completely specific for rCG44606 needs to be identified by tandem mass spectrometry to prove the protein spot identity as rCG44606. CK plays central roles in energy transduction in tissues with large energy demand and fluctuation, such as heart. CK can reversibly catalyze the transfer of phosphate between ATP and various phosphogens. The mitochondrial creatine kinase (CKm) exists in the mitochondrial intermembrane space, where it produces PCr from ATP and creatine (ATP + creatine → ADP + phosphocreatine (PCr)). PCr is an energy buffer and intermediate state form of energy; it is the messenger between subcellular sites of ATP production and those of energy utilization (Wallimann et al., 1992). Clinically, CK can be assayed in blood as a marker of myocardial infarction.

**Table 2:** NCBI blast comparison between rCG44606 and creatine kinase *S*-type.

NCBI blasted proteins	Identities^a^	Positives^b^	Gaps^c^
rCG44606	227/247(92%)	233/247(94%)	9/247(3%)
Creatine kinase *S*-type, mitochondrial precursor (Rattus norvegicus)			

**Notes.**

a The identical amino acid rate between rCG44606 and creatine kinase *S*-type, mitochondrial (Rattus norvegicus).

b The similarity rate of amino acid pairs between rCG44606 and creatine kinase *S*-type, mitochondrial (Rattus norvegicus).

c The amino acid number of deletions and insertions in a protein sequence.

GRP75 (glucose-regulated protein75, also called mortalin or mthsp70) up-regulated more than 2-fold when mitoKATP was blocked. It is a stress protein which belongs to the heat shock protein 70 (HSP70) family (Wadhwa et al., 1993). It not only has general cytoprotective effects against stressful conditions such as glucose deprivation (GD) and oxidative injury (Liu et al., 2005; Yang et al., 2008), but also plays a role in mitochondrial protein importing and folding (Geissler et al., 2001). Paillard et al. (2013) found that inhibition of Grp75 decreased protein interaction within the CypD/VDAC1/Grp75/IP3R1 complex, which attenuated mitochondrial Ca^2+^ overload and protected cardiomyocytes from hypoxia-reoxygenation. Liu et al. (2015) reported that when the mitochondria of SH-SY5Y cells were impaired by A53T *α*-syn, expression of GRP75 decreased significantly. Furthermore, down-regulation of GRP75 increased mitochondrial dynamics by virtue of decreasing *α*-syn’s translocation to mitochondria. They suspected that a compensatory mechanism of GRP75 was implicated in the pathogenesis of Parkinson’s disease. In the present study, the expression of the “bad” protein was doubled in 5-HD group, where the mitoKATP was blocked before reperfusion. Further studies examining the expression change of GRP75 on MIRI are merited.

MICOS complex subunit Mic60 (IMMT) upregulated 2.3 times when mitoKATP was blocked. Whether expression change of IMMT affects MIRI is a puzzle. IMMT is a component of MICOS, a large protein complex locates at mitochondrial inner membrane and plays crucial roles in the crista junctions’ and inner membrane architecture’s maintenance as well as the formation of contact sites to the outer membrane (http://www.uniprot.org/uniprot/Q3KR86).

Five of the differential proteins came from IPo vs. 5-HD. mitoKATP blockade resulted in the upregulation of IMMT and GRP75 but the down-regulation of DLDH, ATPA and rCG44606. These results indicate that mitoKATP opening take part in the proteomic anti-MIRI effect of IPo. This trend is in accordance with previous reports (Gross, 1995; Jin et al., 2012; Mykytenko et al., 2008) and our study (Cao et al., 2015). Using digital gene expression profiling (DGE), we detected that mitoKATP opening (with diazoxide) or closing (with 5-HD) in A/R treated adult rat cardiomyocytes significantly affected three energy metabolism related genes (*Idh2*, *MT-ND6*, and *Acadl*). Bioinformatics analysis detected 20 differential genes that were enriched in energy metabolism related pathways. Afterwards, we confirmed that mitoKATP opening increased ATP content in the adult cardiomyocytes at the end of reoxygenation (Cao et al., 2015).

Collectively, our data suggest some of these differential proteins, such as DLD, ATPA, NDUFV1 and SDHA, could be potential therapeutic targets to mitigate malfunction of ETC, maintain a homeostasis of energy metabolism and defeat MIRI. Under the circumstance of ischemia, energy is desperately needed, but its supply reduces rapidly in ischemic cardiomyocytes (Arslan et al., 2013). Strategies that aim at increasing energy supply or modulating energy expenditure in myocardial I/R process may be promising approaches to reduce MIRI (Arslan et al., 2013; Cao et al., 2015). For example, Chinese traditional medicine QiShenYiQi Pills protect heart against myocardial I/R injury by modulating energy supply (Lin et al., 2013). Fibroblast growth factor (FGF)-21 protects H9c2 cells against I/R injury mainly through recovery of the energy supply (Wei-Tao et al., 2013). Future research incorporating the gain- and/or loss-of-function approaches for the ETC components or some of the above mentioned proteins should be helpful to assure their specific effects and to determine relevance to IPo during MIRI. The microRNA-, lentivirus- or exosome-based gene (including mtDNA) manipulation strategies may be effective in interfering expression of ETC components. In addition, indirect regulation of their activity is an alternative choice. For example, proteomic changes in I/R mouse hearts could be reversed or circumvented by proteins found in mesenchymal stem cell (MSC)-derived exosomes (Lai et al., 2013), intravenously given a bolus of MSC-derived exosomes effectively reduced infarct size by 45% (Arslan et al., 2013). Indirectly modulating ETC activity with STAT3 also presented cardioprotective outcome (Szczepanek et al., 2015). DLA (3,4-dihydroxyl-phenyl lactic acid) ameliorated cardiac reperfusion injury by the indirect prevention of the decrease of NDUFA10 (one subunit of complex I) (Yang et al., 2015).

Several limitations exist in our study. We used narrow pH (5–8) IPG strips, which would had resulted in some undetected differential proteins, for a number of subunits of ETC have an isoelectic point at the range of pH 8–11.5. Separating these hydrophobic membrane proteins in the basic pH range is difficult but pI values of some differential proteins may be more than 8. This may be the reason that only eight differential proteins were found in present study. A wider pH range strips, e.g., NL pH 3–11 or additional pH 7–11 IPG strips could be better choices. It should be also noted that although PMF was commonly used for protein identification as we and other researchers used (Kim et al., 2006; Di Stadio et al., 2016), tandem mass spectrometry could be an alternative approach for measuring tryptic digests on a MALDI TOF/TOF system especially in the case of rCG44606.

## Supplemental Information

10.7717/peerj.1706/supp-1 Data S1 Raw data

10.7717/peerj.1706/supp-2 Supplemental Information 1 MASCOT search results

## Additional Information and Declarations

### Data Availability

The following information was supplied regarding data availability: The research in this article did not generate any raw data.
